# Triple return on investment: the cost and impact of 13 interventions that could prevent stillbirths and save the lives of mothers and babies in South Africa

**DOI:** 10.1186/s12884-015-0456-9

**Published:** 2015-02-18

**Authors:** Julia Michalow, Lumbwe Chola, Shelley McGee, Aviva Tugendhaft, Robert Pattinson, Kate Kerber, Karen Hofman

**Affiliations:** Priority Cost-Effective Lessons for Systems Strengthening-South Africa (PRICELESS SA), Medical Research Council/Wits Rural Public Health and Health Transition Research Unit (Agincourt), Johannesburg, South Africa; School of Public Health, Faculty of Health Sciences, University of the Witwatersrand, 27 St Andrews Road, Parktown, 2193 Johannesburg, South Africa; Department of Obstetrics and Gynecology, Medical Research Council Maternal and Infant Health Care Strategies Research Unit, University of Pretoria, Pretoria, South Africa; Save the Children, Cape Town, South Africa

**Keywords:** Stillbirths, Maternal health, Child health, Cost analysis, South Africa

## Abstract

**Background:**

The time of labor, birth and the first days of life are the most vulnerable period for mothers and children. Despite significant global advocacy, there is insufficient understanding of the investment required to save additional lives. In particular, stillbirths have been neglected. Over 20 000 stillbirths are recorded annually in South Africa, many of which could be averted. This analysis examines available South Africa specific stillbirth data and evaluates the impact and cost-effectiveness of 13 interventions acknowledged to prevent stillbirths and maternal and newborn mortality.

**Methods:**

Multiple data sources were reviewed to evaluate changes in stillbirth rates since 2000. The intervention analysis used the Lives Saved tool (LiST) and the Family Planning module (FamPlan) in Spectrum. LiST was used to determine the number of stillbirths and maternal and neonatal deaths that could be averted by scaling up the interventions to full coverage (99%) in 2030. The impact of family planning was assessed by increasing FamPlan’s default 70% coverage of modern contraception to 75% and 80% coverage. Total and incremental costs were determined in the LiST costing module. Cost-effectiveness measured incremental cost effectiveness ratios per potential life years gained.

**Results:**

Significant variability exists in national stillbirth data. Using the international stillbirth definition, the SBR was 17.6 per 1 000 births in 2013. Full coverage of the 13 interventions in 2030 could reduce the SBR by 30% to 12.4 per 1 000 births, leading to an MMR of 132 per 100 000 and an NMR of 7 per 1 000 live births. Increased family planning coverage reduces the number of deaths significantly. The full intervention package, with 80% family planning coverage in 2030, would require US$420 million (US$7.8 per capita) annually, which is less than baseline costs of US$550 million (US$10.2 per capita). All interventions were highly cost-effective.

**Conclusion:**

This is the first analysis in South Africa to assess the impact of scaling up interventions to avert stillbirths. Improved coverage of 13 interventions that are already recommended could significantly impact the rates of stillbirth and maternal and neonatal mortality. Family planning should also be prioritized to reduce mortality and overall costs.

## Background

Despite increasing global attention to maternal, newborn and child health, issues surrounding stillbirths remain largely neglected. Stillbirths were not included in tracking progress on the Millennium Development Goals (MDGs) and have received little recognition for intervention strategies and programs globally; stillbirth targets are absent from the Sustainable Development Goals. Stillbirths are however one of the most adverse outcomes of pregnancy, causing significant negative social and health implications for families and society [1].

In 2009, 2.6 million third-trimester stillbirths were estimated worldwide, of which 98% occurred in low and middle-income countries [2]. Over 55 stillbirths occur daily in South Africa and the 2013 institutional third trimester stillbirth rate (SBR) was 17.6 per 1 000 births [3]. This far exceeds the SBRs in other middle-income countries such as Brazil, Russia and China (all approximately 10 per 1 000 births) [2]. Unlike most low and middle-income countries (LMICs), South Africa does include stillbirths in the reporting of vital statistics. The Cause of Death Certification (DHA-1663) registers stillbirths and neonatal deaths occurring in both public and private sector facilities [4,5]. Data are registered with the Department of Home Affairs and analyzed by Statistics South Africa (Stats SA). Public sector data are collected by the District Health Information System (DHIS) and the Perinatal Problem Identification Program (PPIP). PPIP is an electronic audit of perinatal and maternal deaths implemented in 588 facilities in South Africa (representing 73% of institutional births recorded by the DHIS) [6]. Given that most South African women (approximately 90%) give birth in health facilities [7], the number of stillbirths occurring at home is likely small. Although these mechanisms are in place for capturing stillbirth statistics, records are often incomplete due to under-reporting and delayed or late registration. There is also evidence for misclassification of causes [8].

Within the ICD-10 classification, stillbirths are defined by a birthweight of 500 g or more, or if birthweight is unknown, gestational age of 22 completed weeks or more, or crown-heel length greater than 25 cm. For international comparison, WHO recommends a third trimester stillbirth definition where birthweight is above 1 000 g, gestational age is 28 complete weeks or more, or crown-heel length is greater than 35 cm [4]. In LMICs, stillbirths are often not weighed, making it difficult to differentiate between second and third trimester deaths [9,10].

The causes of stillbirths are closely connected to those of maternal and neonatal morbidity and mortality. Focused attention on stillbirth prevention could thus also lead to a reduction in maternal and newborn deaths. Distinguishing between stillbirths that occur during the antepartum (before the onset of labor) and intrapartum (during labor and birth) periods has important programmatic implications. The time of death is however not always known, so the presence or absence of maceration is used as a surrogate marker for antepartum and intrapartum death, respectively. Maceration implies that death occurred more than 12 hours before delivery [11]. In South Africa, nearly two-thirds of stillbirths occur during the antepartum period [6] and while the cause of almost 50% of antepartum deaths is unknown, the remainder could be prevented with improved periconceptual and antenatal care [12]. Maternal risk factors for stillbirths are hypertension, antepartum hemorrhage, and spontaneous preterm labor (Table 1). Hypertensive disorders (19%) account for the greatest proportion of antepartum stillbirths while intrapartum asphyxia (29%) and antepartum hemorrhage (27%) account for the greatest number of intrapartum stillbirths.

**Table 1:** **Causes of third trimester stillbirth in South Africa**

**Cause**	**Fresh stillbirths (%)**	**Macerated stillbirths (%)**
Intrapartum asphyxia	28.9	4.6
Antepartum hemorrhage	26.9	9.1
Unexplained intrauterine death	15.7	47.9
Hypertensive disorders	10.1	19.1
Spontaneous preterm labor	7.2	4.2
Fetal abnormality	4.4	2.3
Infections	2.3	3.9
Intrauterine growth retardation	1.3	2.9
Maternal disease	1.2	3.6
Miscellaneous	0.9	1.3
No obstetric cause/Not applicable	0.6	0.6
Trauma	0.5	0.4
Total	100	100

Source: [6].

The World Health Organization (WHO) has stressed the importance of ending these preventable deaths. The Every Newborn action plan launched in 2014 sets a target to reduce stillbirth rates globally to 10 per 1 000 births by 2035, with an interim goal of 12 per 1 000 births by 2030 [13]. While South Africa is a signatory to the plan and has set a national target that is more ambitious than the global one (reduce stillbirth rate to 10 per 1 000 births by 2016) [14], the country is not currently making progress towards meeting these goals.

Given the need for high quality, local data as a basis for priority setting and budgeting [15], our analysis (1) reviews available South African data to approximate trends in stillbirth rates between 2000 and 2013 and to investigate the national distribution of stillbirths; and (2) evaluates the costs of 13 priority interventions and their impact on stillbirths and maternal and newborn mortality in South Africa between 2015 and 2030. The paper predicts the potential numbers of deaths averted and estimates the resources required to scale up these interventions.

## Methods

### Stillbirth trends in South Africa

Data on stillbirths in South Africa were reviewed to evaluate changes in stillbirth rates over time. Stats SA, DHIS and PPIP data were collated to approximate trends in South Africa between 2000 and 2013. Stats SA data were obtained from a report by the Medical Research Council (MRC) [16]. The data have been adjusted for under-registration. DHIS data were acquired from the South African Health Review [3,17] and PPIP data were compiled from Saving Babies reports [6,18-23]. Additional PPIP data were made available to the authors for 2012 and 2013, which were used to approximate the distribution of stillbirths in health facilities in South Africa.

### Projection of priority interventions

The Lives Saved Tool (LiST) was used to analyze the number of stillbirths and deaths of mothers and newborns that could be averted by scaling up the intervention packages for maternal and neonatal health to full coverage (99%). LiST is a module in Spectrum, a demographic software package, which preloads national data for health status, mortality rates, and coverage of more than 60 interventions and their effectiveness in relation to specific causes of death [24,25]. The modelling methods in LiST have been widely reviewed [26,27]. The effects of scaling up the coverage of specific interventions were modelled to estimate the deaths averted, overall and by each intervention. The analysis was performed using Spectrum version 5.04.

The WHO third trimester stillbirth definition (>1 000 g) was used to ensure projections are of international relevance. The modelled interventions would however also be effective in averting second trimester stillbirths (500–1 000 g).

**Figure 1:**
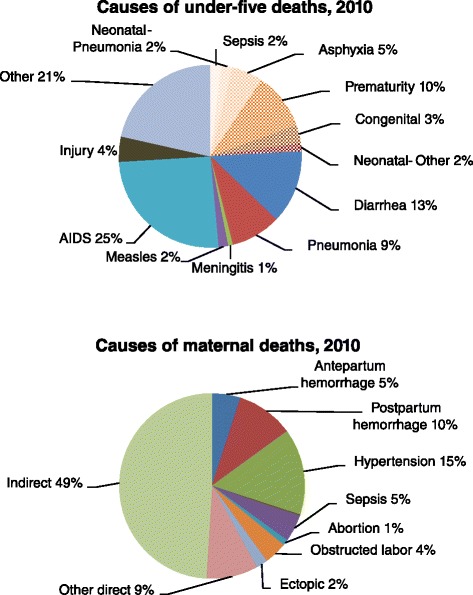
**Causes of newborn, child and maternal deaths in South Africa used in the LiST model.** Source: Adapted from Medical Research Council burden of disease estimates in 2010.

Mortality rates were estimated for 2012, or the closest year: third trimester stillbirth rate (SBR) was 17.6 per 1 000 births [6], maternal mortality ratio (MMR) was 269 deaths per 100 000 live births [28] and neonatal mortality rate (NMR) was 12 per 1 000 live births [28]. The causes of maternal [29] and newborn [30] mortality were adapted from the South African Medical Research Council Burden of Disease (BOD) estimates to fit the causal categories provided by LiST (Figure 1). The causes of death categorized in LiST are slightly different from those presented by the MRC. For example, neonatal diarrhoea is not reported separately in the MRC BOD, but rather combined with under-five diarrhoeal deaths. Therefore, we separated these using the default proportions in LiST. LiST broadly defines stillbirths as antepartum or intrapartum. Rates of 35% for intrapartum and 65% for antepartum stillbirths were calculated by dividing the numbers of fresh and macerated stillbirths by the total number of stillbirths, respectively, using data provided by PPIP for 2012 and 2013 [6].

In *The Lancet’s* Stillbirth Series, Bhutta *et al.* [31] identified a package of 10 interventions during pregnancy and childbirth to prevent stillbirths globally, including expanded antenatal care packages for improved detection and management of hypertension and diabetes in pregnancy, improved detection of high risk pregnancies and fetal compromise, and induction of labor after 41 weeks gestation. Pattinson *et al.* [32] include five additional interventions in their global analysis which provide significant health benefits for mothers and newborns. These interventions were adapted for the South African context. Interventions for malaria prevention were excluded as South Africa has succeeded in preventing malaria transmission throughout most of the country following introduction of DDT in 2001 [33]. Folic acid supplementation or fortification was also excluded because South Africa’s folic acid fortification program, implemented in 2003, has achieved significant declines in neural tube defects [34]. Early detection and treatment of HIV in pregnant mothers was added to the model as a maternal intervention, since over 40% of maternal deaths in South Africa are due to AIDS [29]. The availability of obstetric care was also adjusted. The obstetric levels in LiST must sum to cover 100% of deliveries. This includes assisted and unassisted home deliveries and facility deliveries with essential care, basic emergency obstetric care (EmOC) and comprehensive EmOC. At baseline, facility delivery coverage is distributed between the three levels of care. In an ideal scenario, everyone in South Africa needing comprehensive EmOC would have access to it. This intervention has thus been scaled up to 99% and the other obstetric packages have correspondingly been scaled down to maintain 100% delivery coverage.

Table 2 presents the final 13 priority interventions, along with their effect estimates and affected fractions. In *The Lancet’s* Stillbirth Series, the intervention effects were compiled using the literature review criteria developed by the Child Health Epidemiology Group (CHERG) [35] and through Delphi consultation with global experts and practitioners [31,32]. The same effects have been used in this analysis; these values are different from the default effects in version 5.04 of LiST, except where indicated. The effects used are also available online as supplementary documentation for LiST [36]. In keeping with *The Lancet’s* methods, pathways were added to LiST for the effect of hypertensive disease case management on stillbirths and the effect of labor and delivery management on maternal sepsis, neonatal sepsis and neonatal tetanus [32]. The affected fractions were also derived from the online supplementary material [36]. It has been indicated where default affected fractions were used. South African experts were consulted to determine the effect and affected fraction for the added intervention, early detection and treatment of HIV in pregnant mothers.

**Table 2:** **Effect and affected fraction estimates for interventions to reduce stillbirths and maternal and neonatal deaths**

	**Stillbirths**		**Maternal deaths**		**Neonatal deaths**	
	**Effect**	**AF**	**Effect**	**AF**	**Effect**	**AF**
**Interventions for stillbirth prevention**						
Syphilis detection and treatment	0.82 for antepartum stillbirths*	0.21*	Minimal effect	-	0.03 for sepsis	1
Hypertensive disease case management	0.20 for antepartum and intrapartum stillbirths	1	0.59 for hypertensive disease	1*	Effect calculated as part of BEmOC and CEmOC	
Diabetes case management	0.10 for antepartum and intrapartum stillbirths*	0.106*	Unknown effect	-	Unknown effect	-
MgSO4 Management of pre-eclampsia	0.20 for antepartum and intrapartum stillbirths*	0.213*	0.59 for hypertensive disease*	1*	Unknown effect	-
Fetal growth restriction detection and management	0.20 for antepartum and intrapartum stillbirths*	0.202*	Unknown effect	-	Unknown effect	-
Labor and delivery management: Essential obstetric care	0.23 for intrapartum stillbirths	1*	0.10 for sepsis	1*	0.25 for asphyxia*; 0.10 for prematurity*; 0.25 for sepsis; 0.36 for tetanus	1*
Labor and delivery management: Basic emergency obstetric care (BEmOC)	0.45 for intrapartum stillbirths	1*	0.08 for obstructed labor; 0.20 for antepartum hemorrhage*; 0.65 for postpartum hemorrhage; 0.50 for sepsis	1*	0.40 for asphyxia*; 0.10 for prematurity*; 0.25 for sepsis; 0.36 for tetanus	1*
Labor and delivery management: Comprehensive emergency obstetric care (CEmOC)	0.75 for intrapartum stillbirths	1*	0.99 for obstructed labor; 0.80 for antepartum hemorrhage*; 0.95 for postpartum hemorrhage; 0.99 for hypertensive disease of pregnancy; 0.70 for sepsis	1*	0.80 for asphyxia*; 0.10 for prematurity*; 0.25 for sepsis; 0.36 for tetanus	1*
Induction of labor for pregnancies lasting 41+ weeks (CEmOC only)	0.69 for antepartum and intrapartum stillbirths*	0.036*	No effect	-	Unknown effect	-
**Interventions for maternal and newborn health**						
Early detection and treatment of HIV in pregnant women	Unknown effect	-	0.90 for other indirect causes	0.8	Unknown effect	-
Tetanus toxoid immunization during pregnancy	No effect	-	0.98*	0.005*	0.94 for tetanus*	1*
Antibiotics for preterm premature rupture of membranes	Unknown effect	-	0.26 for sepsis	0.1	0.12 for prematurity*; 0.08 for sepsis	1 for both
Antenatal corticosteroids for preterm labor	No effect	-	No effect	-	0.53 for prematurity*	1
Active management of the third stage of labor	No effect	-	0.27 for postpartum hemorrhage	1*	No effect	-
Neonatal resuscitation	No effect	-	No effect	-	0.3 for asphyxia*; 0.1 for prematurity*	1 for both*

AF = Affected fraction; *Default LiST value in Spectrum V5.04. Sources: Adapted from Bhutta [31,32,36].

A one day expert consultation was hosted locally to discuss trends in maternal and newborn interventions in South Africa. Twenty-three participants were invited from the health sector, including clinicians, academics and those in positions at national and district level. Coverage levels for all interventions were reviewed and modified by the expert panel (Table 3). Unchanged default LiST coverage levels have been indicated. A linear increase in coverage was applied to each of the 13 priority interventions from 2014, the baseline coverage year, to reach 99% coverage in 2030. Coverage levels for other maternal and child health interventions were not altered, so that the overall impact of the priority interventions on South Africa’s SBR, MMR and NMR could be isolated. (Ramping up the coverage of all maternal, newborn and child interventions simultaneously in LiST did not have a significant impact on the deaths averted by the 13 interventions). In order to evaluate the impact of increasing the modern contraceptive prevalence rate (CPR) on pregnancies and subsequent stillbirths and maternal and newborn mortality, three scenarios were analyzed for family planning using the FamPlan module available in Spectrum: 70% coverage in 2030 (FamPlan default); scale up to 75% coverage in 2030; and scale up to 80% coverage in 2030. For all three scenarios, a baseline family planning coverage of 65% and a total fertility rate (TFR) of 2.81 were used (default FamPlan values).

**Table 3:** **Baseline coverage estimates for interventions to reduce stillbirths and maternal and neonatal deaths**

**Interventions**	**Baseline 2014 coverage (%)**
**Interventions for stillbirth prevention**	
Syphilis detection and treatment	92
Hypertensive disease case management	40
Diabetes case management	10
MgSO4 Management of pre-eclampsia	75
Fetal growth restriction detection and management	10
Labor and delivery management	
Essential care	22*
Basic emergency obstetric care	13*
Comprehensive emergency obstetric care	53*
Induction of labor for pregnancies lasting 41+ weeks	10*
**Interventions for maternal and newborn health**	
Early detection and treatment of HIV in pregnant women	40
Tetanus toxoid immunization during pregnancy	77*
Antibiotics for preterm premature rupture of membranes	25
Antenatal corticosteroids for preterm labor	20
Active management of the third stage of labor	80
Neonatal resuscitation	40*

*Default LiST value in Spectrum V5.04. Source: Consultation with expert South African panel.

### Intervention cost-effectiveness

The interventions were costed using the costing module in LiST, using the most recently available data. In the model, the cost of each intervention is based on four components: personnel and labor; drugs and supplies; other recurrent costs; and capital costs. Default international prices were used for drugs and supplies but staff remuneration was added based on public health salary data for South Africa [37,38]. The direct (recurrent) and indirect (capital) costs for hospitalization and outpatient visits were obtained from the WHO-CHOICE (CHOosing Interventions that are Cost Effective) database [39]. Family planning was costed by combining the costs for each contraceptive method, including oral contraceptive pills, condoms, injectables, intrauterine devices, and female and male sterilization. Costs related to infrastructure development are not included in LiST [40]. All costs were adjusted to 2014 US dollars. All per capita estimations used a population size of 54 million, according to the Stats SA mid-year population estimates for 2014 [41].

Cost-effectiveness was determined by combining the effects (additional lives saved) and costs calculated by LiST. The baseline used was 2014 such that effects and incremental costs were calculated based on the difference between estimates in 2014 and 2030. Lives saved were multiplied by life expectancy to estimate potential life years gained. A life expectancy at birth of 60 years was used for stillborns and neonates [41] and a Reproductive-Aged Life Expectancy (RALE) [42] of 27 years was used for mothers, based on SA’s 2011 life tables [43]. Incremental cost-effectiveness ratios (ICERs) were obtained by dividing incremental intervention costs by incremental effects. The WHO criteria on cost-effectiveness were used, which specify that an intervention is highly cost-effective if it averts a year of life lost for less than the national gross domestic product (GDP) per capita, cost-effective if 1 to 3 times the GDP per capita, and not cost-effective if greater than 3 times the GDP per capita [44]. Given SA’s GDP per capita (2012) of US$7 500 [45], interventions were considered highly cost-effective if the ICER was less than US$7 500, cost-effective if between US$7 500 and US$22 500, and not cost-effective if more than US$22 500.

Ethical review board approval was not required for this paper as no human subjects were involved and only secondary data were used.

## Results

### Stillbirth trends in South Africa

**Figure 2:**
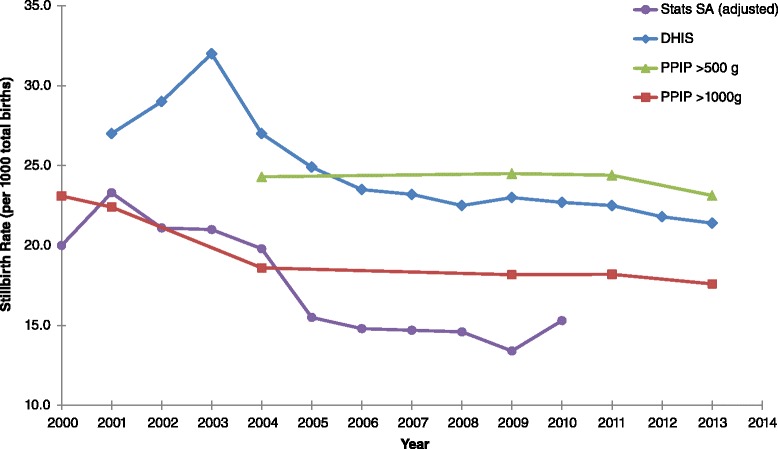
**Stillbirth rates in South Africa (2000 to 2013).** Sources: South African Health Review 2010 [3] and 2012/13 [46]; and Saving Babies reports 2000 – 2014 [6,18-23].

According to the DHIS and PPIP, there are over 20 000 stillbirths recorded in South Africa each year (approximately 55 stillbirths per day) [3,6]. Figure 2 shows the compiled stillbirth rates from 2000 to 2013 for all three data sources. Stats SA and DHIS do not specify a stillbirth definition, although the reported SBRs appear to include all stillbirths (above 500 g). PPIP differentiates between stillbirths weighing more than 500 g and 1 000 g.

The DHIS consistently reports higher stillbirth rates than the vital registration system (Stats SA) [8]. In 2010, the DHIS reported a SBR of 22.7 per 1 000 births while Stats SA reported a SBR of 15.3 per 1 000 births for the same year. In 2011, PPIP however reported a total SBR of 24.4 per 1 000 births (>500 g) and a third trimester SBR of 18.2 per 1 000 births (>1 000 g). In 2013, PPIP reported SBRs of 23.1 (>500 g) and 17.6 (>1 000 g) per 1 000 births [6]. According to the DHIS, the total SBR was 21.4 per 1 000 births in 2013 [3], and this has reduced by 20% since 2001. The PPIP third trimester stillbirths show a reduction of 24% between 2000 and 2013. Similarly, Stats SA data shows a reduction of 24% between 2000 and 2010.

The total SBRs recorded by the DHIS vary among the nine South African provinces [3]. The greatest provincial reductions were achieved in the Eastern Cape (40% reduction to 20.6 per 1 000 births) and the Northern Cape (40% reduction to 26.1 per 1 000 births) between 2001 and 2013, however the SBR in the Northern Cape increased by 20% between 2008 and 2013. The total SBR in South Africa (DHIS data) ranges from a high of 27.4 per 1 000 births in the Free State to a low of 17.4 per 1 000 births in the Western Cape [3]. PPIP reports third trimester SBRs of 21.8 per 1 000 births in the Free State and 12.3 per 1 000 births in the Western Cape [6].

Table 4 highlights the PPIP health facilities where the majority of births and stillbirths occurred in 2012 and 2013. The greatest proportion of stillbirths occurs in district and regional hospitals (76%), where the majority of births occur overall (74%). The highest SBRs however occur in provincial tertiary and national central hospitals, because these facilities manage more complicated cases.

**Table 4:** **National distribution of births and stillbirths by level of facility (2012 and 2013)**

	**CHC**	**DH**	**RH**	**PT**	**NC**	**Total**
Total births	229 933	654 115	386 334	69 470	72 503	1 412 355
Proportion of births (%)	16.3	46.3	27.4	4.9	5.1	100
SBR > 500 g	8.4	20.2	30.2	39.2	42.9	23.1
SBR > 1 000 g	6.2	16.4	22.6	28.8	27.9	17.6
Proportion of stillbirths (%)	5.9	40.5	35.7	8.3	9.5	100

CHC – Community Health Centre; DH – District Hospital; RH – Regional Hospital; PT – Provincial Tertiary Hospital; NC – National Central Hospital; SBR – stillbirth rate per 1 000 births. Source: [6].

### Projection of priority interventions

#### Reduction in maternal and newborn mortality and stillbirth in 2030

If full coverage of the 13 interventions for stillbirths and maternal and newborn health is achieved in South Africa, the model predicts that the SBR could reduce by 30% from 17.6 per 1 000 births in 2014 to 12 per 1 000 births in 2030. The MMR could reduce by 50% to 132 deaths per 100 000 live births and the NMR could reduce by 42% to 7 per 1 000 live births. Reductions in SBR, MMR and NMR are consistent for all three family planning scenarios since the number of births and deaths reduce proportionately.

Approximately 11 600 lives could be saved in 2030, with full coverage of the 13 interventions and 70% coverage of modern contraceptive use. Specifically, 5 400 stillbirths could be averted, and 1 300 maternal lives and 4 800 neonatal lives could be saved per year. When contraception is scaled up to 75% and 80%, the annual number of lives saved reduces to approximately 9 000 and 6 800, respectively.

The number of stillbirths averted and maternal and newborn lives saved reduces due to fewer pregnancies and deaths that occur when family planning coverage is increased. The total number of pregnancies in 2030 reduces from 1.4 million at baseline (2014) to 1.3 million in scenario 1, 1 million in scenario 2 and 770 000 in scenario 3. Correspondingly, the TFR reduces from 2.81 in 2014 to 2.08 in 2030 when the CPR increases to 70% (FamPlan default). Increasing the CPR to 75% and 80% in 2030 results in lower TFRs of 1.61 and 1.21, respectively. This reduction in the number of pregnancies results in fewer deaths. The total number of deaths decreases by 22% when contraceptive prevalence is scaled up to 75% and 42% when the CPR is increased to 80%.

**Figure 3:**
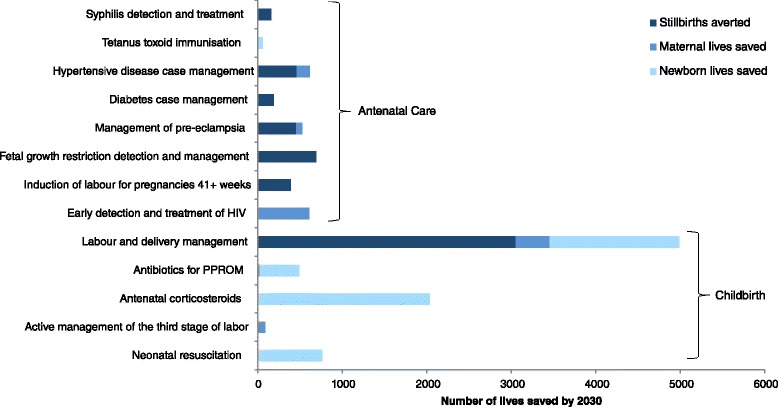
**Number of lives saved in 2030 with full intervention coverage and 70% family planning coverage.**

Of the stillbirths averted, 72% resulted from intrapartum interventions and 28% from antenatal interventions. Labor and delivery management has the most significant effect; it accounts for approximately 60% of stillbirths averted and 40% of lives saved overall (Figure 3). Of the antenatal care interventions, fetal growth restriction detection and management and hypertensive disease case management have the greatest impact in reducing stillbirth rates.

### Costs and cost-effectiveness

The baseline cost in 2014 for the 13 interventions was estimated at US$330 million (about US$6.2 per capita). This includes US$290 million for stillbirth specific interventions and US$35 million for maternal and newborn health interventions. If these interventions were scaled to full coverage, with the default family planning scale up of 70%, the total cost of these interventions would be US$380 million (US$7 per capita) in 2030. When the CPR is increased to 75% and 80%, the total costs in 2030 reduce to US$300 million (US$5.7 per capita) and US$230 million (US$4.3 per capita), respectively.

Table 5 presents the additional costs, effects and cost-effectiveness of interventions for stillbirths and maternal and newborn health with default family planning scale up. All of the interventions are highly cost effective (ICERs below US$7 500). Among the interventions for stillbirths, hypertensive disease case management has the lowest ICER (US$1) while diabetes case management has the highest (US$746). Neonatal resuscitation has the lowest ICER (US$2) among the interventions with specific impact for mothers and newborns. The most additional resources are required for labor and delivery management, at approximately US$20 million.

**Table 5:** **Projected incremental costs and effects in 2030 with family planning at 70%**

	**Additional lives saved**			**Incremental costs (US$)**	**Life years gained**	**Incremental cost (US$)/LY gained**
	**Stillbirth**	**Maternal**	**Neonatal**			
**Interventions for stillbirth prevention**						
Syphilis detection and treatment	160	-	0	106 550	9 600	11
Hypertensive disease case management	460	150	-	35 040	31 650	1
Diabetes case management	180	-	-	8 194 520	10 800	759
MgSO4 Management of pre-eclampsia	450	70	-	5 492 600	28 890	190
Fetal growth restriction detection and management	690	-	-	2 876 410	41 400	69
Labor and delivery management	3 050	400	1 540	19 447 320	286 200	68
Induction of labor for pregnancies lasting 41+ weeks	390	-	-	2 436 610	23 400	104
**Interventions for maternal and newborn health**						
Early detection and treatment of HIV	-	610	-	10 930 280	16 470	664
Tetanus toxoid immunization	-	2	50	614 240	3 054	201
Antibiotics for PPROM	-	20	470	769 780	28 740	27
Antenatal corticosteroids	-	-	2 030	4 527 270	121 800	37
Active management of the third stage of labor	-	80	-	1 547 670	2 160	717
Neonatal resuscitation	-	-	760	81 670	45 600	2
**Total**	**5 380**	**1 332**	**4 850**	**57 059 960**	**649 764**	**88**

US$ = United States Dollars; numbers rounded to nearest 10 to indicate uncertainty.

When family planning is scaled up, fewer pregnancies result in lower rates of morbidity and mortality and there are thus fewer mothers and newborns requiring medical interventions. The incremental costs are therefore lower for higher CPRs, as shown in Table 6. Significant cost reductions are observed for syphilis detection and management, management of pre-eclampsia, labor and delivery management, tetanus toxoid immunization and active management of the third stage of labor. The costs for diabetes case management and fetal growth restriction detection and management remain high.

**Table 6:** **Projected incremental costs and effects in 2030 for each family planning scenario**

	**Family planning at 70%**		**Family planning at 75%**		**Family planning at 80%**	
	**Number of lives saved**	**Incremental costs (US$)**	**Number of lives saved**	**Incremental costs (US$)**	**Number of lives saved**	**Incremental costs (US$)**
**Interventions for stillbirths**						
Syphilis detection and treatment	160	106 550	120	−2 544 770	90	−4 794 400
Hypertensive disease case management	610	35 040	470	21 240	360	9 520
Diabetes case management	180	8 194 520	140	7 932 270	110	7 694 400
MgSO4 Management of pre-eclampsia	520	5 492 600	410	−917 110	310	−6 355 730
Fetal growth restriction detection and management	690	2 876 410	530	2 784 350	400	2 700 860
Labor and delivery management	4990	19 447 320	3 870	−41 901 940	2 920	−93 956 580
Induction of labor for pregnancies lasting 41+ weeks	390	2 436 610	300	1 856 720	230	1 364 690
**Interventions for maternal and newborn health**						
Early detection and treatment of HIV	610	10 930 280	470	2 462 090	360	−4 705 650
Tetanus toxoid immunization	52	614 240	40	−193 090	30	−878 110
Antibiotics for PPROM	490	769 780	380	542 300	280	349 290
Antenatal corticosteroids	2030	4 527 270	1 580	3 269 430	1 190	2 202 160
Active management of the third stage of labor	80	1 547 670	70	25 240	50	−1 266 540
Neonatal resuscitation	760	81 670	590	51 850	440	26 330
**Total**	**11 562**	**57 059 960**	**8 970**	**−26 611 420**	**6 770**	**−97 609 760**

US$ = United States Dollar; numbers rounded to nearest 10 to indicate uncertainty.

The costs for family planning increase with higher contraceptive prevalence. The baseline cost in 2014 is US$140 million (US$2.6 per capita). In 2030, the total costs increase to US$167 million when the CPR is 70% (US$3.1 per capita), US$181 million (US$3.4 per capita) when the CPR is 75% and US$193 million (US$3.6 per capita) when the CPR is 80%.

Table 7 summarizes the intervention costs, family planning costs and the combined overall costs for each scenario in 2030. The overall costs reduce as family planning coverage increases, despite the higher costs incurred for family planning. When the CPR is 80%, the total cost in 2030 is US$420 million (US$7.8 per capita). The corresponding overall incremental cost of -US$44 million (−US$0.8 per capita) is negative to indicate that the cost in 2030 is less than that of the baseline year (2014).

**Table 7:** **Summary of projected total and incremental costs in 2030 for each family planning scenario**

	**Family planning at 70%**		**Family planning at 75%**		**Family planning at 80%**	
	**Total cost (US$)**	**Incremental cost (US$)**	**Total cost (US$)**	**Incremental cost (US$)**	**Total cost (US$)**	**Incremental cost (US$)**
Intervention costs	383 238 300	57 059 960	299 566 910	−26 611 430	228 568 580	−97 609 750
Family planning costs	166 992 040	27 368 480	180 953 420	41 329 860	192 830 510	53 206 950
Overall cost	550 230 340	84 428 440	480 520 330	14 718 430	421 399 090	−44 402 800
Overall cost per capita	10.2	1.6	8.9	0.3	7.8	−0.8

US$ = United States Dollars; numbers rounded to nearest 10 to indicate uncertainty.

## Discussion

This is the first analysis in South Africa to assess the impact of scaling up interventions to avert stillbirths during pregnancy and childbirth. Our model shows that scaling up 13 essential interventions could prevent a significant percentage of stillbirths and save the lives of mothers and newborns. If full coverage (99%) is achieved in 2030, these interventions could reduce the SBR by 30% from 17.6 per 1 000 births in 2014 to 12.4 per 1 000 births, and could lead to an MMR of 132 per 100 000 live births and an NMR of 7 per 1 000 live births. This would lead to approximately 6 200 additional maternal and newborn lives saved and approximately 5 400 stillbirths averted in 2030. These estimates are made with the contraceptive prevalence rate for modern family planning methods set to 70% in 2030. When CPR is increased to 75% and 80%, the model predicts that 9 000 lives and 6 800 lives could be saved, respectively, due to the reduced number of pregnancies and deaths.

The majority of stillbirths averted are during the intrapartum period (72%), despite higher rates of antepartum stillbirth at baseline. This occurs for several reasons. Causes of intrapartum stillbirth are better understood and documented, and have proven solutions. Unexplained intrauterine death is recorded for 48% and 16% of antepartum and intrapartum stillbirths, respectively [6]. These unidentified causes make it difficult to implement a comprehensive set of interventions during the antenatal period. As a result, the antenatal interventions which have been modelled are effective in small subsets of pregnant women and have effectiveness values that range from 10% for diabetes case management to 20% for management of pre-eclampsia, hypertensive diseases, and fetal growth restriction. While syphilis detection and treatment has an effect size of 82%, South Africa already has high coverage of syphilis detection and treatment in 2014 (92%), thereby reducing the incremental impact of scaling up this intervention.

Labor and delivery management has the most significant effect on stillbirth outcomes. Comprehensive emergency obstetric care for women delivering in facilities includes augmentation of labor, instrumental delivery and emergency caesarean section. Despite having high levels of facility deliveries and skilled birth attendance in South Africa, many women and babies die during pregnancy and childbirth due to delays in accessing services and poor quality of care once in health facilities [47]. This has not been represented in LiST, since the model relates the quality of an intervention to its level of coverage; it is assumed that labor and delivery management is delivered at high quality due to its high baseline coverage.

With full intervention coverage and family planning at 80%, the package to reduce stillbirths, maternal and newborn deaths is estimated to cost US$420 million (US$7.8 per capita) in 2030. This cost is less than the projected baseline cost in 2014. The intervention for labor and delivery management requires the most additional resources (US$20 million). The intervention costs increase with reduced family planning coverage. When family planning coverage is 70% in 2030, the overall cost increases to US$550 million (US$10.2 per capita), requiring an additional investment of US$84 million (US$1.6 per capita). The interventions are all highly cost-effective, given that their ICERs fall well below SA’s GDP per capita of US$7 500.

However, cost calculations do not include the costs of infrastructure development. Taking these into account would increase the total costs. The estimates provided can still guide provincial health department budgetary considerations with regard to providing a maternal and newborn health package.

*The Lancet*’s Stillbirth Series evaluated global rates and causes of stillbirths. It showed that globally, scaling up the initial 15 maternal and neonatal care interventions to full coverage could avert 1.1 million (45%) third-trimester stillbirths, 201 000 (54%) maternal deaths, and 1.4 million (43%) neonatal deaths annually, at an additional cost of US$10.9 billion or $2.32 per person in 2011 [31,32]. Our analysis has also shown a high reduction in mortality, yet the global and country level costs cannot be compared. Costing was additionally performed separately in *The Lancet* analysis and has since been incorporated in LiST [31].

The analysis models an ambitious goal of 99% intervention coverage, which may be difficult for South Africa to accomplish even within the 2030 timeframe. Other interventions such as PMTCT and immunizations have however shown that high coverage levels can be attained with concerted effort. The results provide evidence for the impact of focused attention on stillbirth prevention; South Africa could meet the WHO stillbirth target of 12 per 1 000 births in 2030. This should provide impetus for a renewed focus on stillbirths in the post-2015 development era. South Africa has already shown some commitment to this by setting a national stillbirth rate target [14], consistently monitoring and reporting stillbirths, as well as by endorsing the WHO Every Newborn action plan [13]. Stillbirth targets should also however be reflected in South Africa’s National Development Plan for 2030. The South African National Committee for the Confidential Inquiries into Maternal Deaths (NCCEMD) and the National Perinatal Mortality and Morbidity Committee (NaPeMMCo) have already recommended the interventions in this analysis [8,29]. Considerable effort is however required to ensure these interventions are effectively delivered to those who are currently not receiving them, often the most marginalized and at-risk women and babies. Since the largest proportion of stillbirths occur in district and regional hospitals (76%), efforts should be focused on these facilities where there is greatest potential to save lives.

The coverage of antenatal corticosteroids (ACS) is low at baseline (20%). Currently, ACS are administered primarily in district hospitals but coverage could be even greater if they were promoted at the community health center level. This would allow earlier treatment initiation, prior to transferring the mother to a district hospital. Concern has however been raised over the potentially harmful effects of administering ACS to women not at risk of premature labor and imminent delivery, especially in low income settings [48]. While the skills and resources at the district hospital level enable accurate gestational age estimation and premature labor diagnosis, this may be lacking at the CHC level. Promoting the use of ACS in CHCs should thus be approached with caution in South Africa.

Family planning has been shown to have a significant impact in saving the lives of mothers and babies [49]. Fewer pregnancies in South Africa would also reduce the number of stillbirths. When modern contraceptive prevalence is increased to 80%, the total fertility rate is less than 2 (below the replacement rate), but this is still on par with countries such as Singapore and Brazil [50]. Family planning is crucial for health, development and women’s equality, particularly among high risk groups such as adolescents and HIV-positive women.

The heterogeneity in mortality rate and intervention coverage by province or district is not represented in this national analysis. There is a significant contrast between reported stillbirth rates in the Western Cape and the Free State, for example. Stillbirth reporting is inadequate in some provinces, hence the national SBR is likely an underestimate and the calculated provincial and national SBR reductions are uncertain. Furthermore, PPIP facility coverage has increased significantly between 2000 (27 sites) and 2013 (588 sites). While coverage included each province, the earlier data is less representative.

Barriers to stillbirth reporting are not entirely understood. The formal burial required for registered stillbirths presents a financial disincentive for reporting. Hospital staff may assist families in avoiding this cost by recording stillbirth as a miscarriage [51]. In rural Mpumalanga province, the average funeral cost for children younger than six years is US$116 (adjusted for inflation), which is 92% of the average monthly household income [52]. The definition of stillbirth also affects reporting. Difficulty differentiating between second and third trimester stillbirths, as well as antepartum and intrapartum stillbirths can present additional obstacles. Stats SA accounts for under-reporting by adjusting the reported stillbirth numbers by the same rate of under-reporting in infant deaths. However, it is not known whether the same improvement in infant death registration has been experienced for stillbirths. The decline in stillbirth rates reported by Stats SA may thus result from the adjustment factor [16]. Since the third trimester stillbirth definition was used for modelling and projections, the total number of stillbirths averted is not fully represented.

As the foremost health challenge facing South Africa, prevention and treatment of HIV remains critical at each level of care. The effect of early HIV treatment on maternal deaths has been assessed, yet the model does not consider the impact of HIV/AIDS on stillbirths or newborns. There is some evidence to suggest a relationship between HIV/AIDS and stillbirth outcomes [53-55], but the effects of an HIV/AIDS intervention in this sphere has not been firmly established. Including such effects in this analysis may have had an additional impact on stillbirths in South Africa.

Diabetes case management was the least cost-effective intervention, but the impact of diabetes prevention has not been assessed for stillbirths. With rising obesity rates in South Africa, especially amongst young women [56], weight control before pregnancy would potentially provide a high impact intervention. This has however not yet been documented.

## Conclusion

Each year over 20 000 stillbirths are recorded in South Africa, yet these deaths receive insufficient attention and were not included as a target within the MDGs. South Africa has shown some commitment to reducing stillbirths, as one of the few countries in Africa to consistently record and report stillbirths and by setting a target stillbirth rate within the current national strategic plan [14]. There is however a need for more robust stillbirth data collection to ensure adequate progress tracking. The inter-ministerial committees on maternal, perinatal and child mortality have recognized the importance of focusing on stillbirths and family planning, and have made several recommendations for essential interventions. Family planning is included in the Sustainable Development Goals and can prevent unplanned pregnancies and the subsequent abortions, stillbirths and maternal and newborn deaths. In this analysis we show that focusing on stillbirths with full coverage of 13 essential interventions for mothers and newborns in 2030 could reduce the SBR to 12 per 1 000 births, and could result in an MMR of 132 per 100 000 live births and an NMR of 7 per 1 000 live births. If the coverage of family planning was simultaneously increased to 80% in 2030, the full package would cost US$7.8 per capita (less than the baseline cost in 2014). The costs appear to be substantial, but are within the capacity of the South African health budget. The cost-effectiveness values are also below the recommended willingness-to-pay threshold of the South African per capita GDP, implying that even at scale, these interventions could be affordable. In South Africa’s post-2015 agenda, investing in family planning and the 13 essential interventions will have a triple return in preventing stillbirths and saving the lives of mothers and newborns.
